# Obstacles and sustainability of enhanced recovery after surgery in pediatric laparoscopic pyeloplasty

**DOI:** 10.3389/fped.2024.1437262

**Published:** 2024-12-03

**Authors:** Wenliang Zhu, Huajian Lai, Ziqin He, Yifei Zhang, Qiang Guo, Wenwen Zhong, Lei Ye, Jianguang Qiu, Dejuan Wang

**Affiliations:** ^1^ Department of Urology, The Sixth Affiliated Hospital, Sun Yat-sen University, Guangzhou, China; ^2^ Department of General Surgery, Guangdong Provincial People’s Hospital’s Nanhai Hospital, Foshan, China

**Keywords:** ERAS, laparoscopic pyeloplasty, UPJO, pediatric, obstacles

## Abstract

**Objectives:**

Previous studies on Enhanced Recovery After Surgery (ERAS) in pediatric Laparoscopic Pyeloplasty (LP) lacked clear control cases and discussed the obstacles in the implementation process. This article details the obstacles and lessons learned during the implementation of ERAS in patients with ureteropelvic junction obstruction (UPJO).

**Methods:**

An ERAS protocol was implemented in the UPJO population undergoing LP, which included preoperative, intraoperative, and postoperative management. The clinical data of ERAS program Before Implementation (BI) and After Implementation (AI) were collected and analyzed retrospectively.

**Results:**

A total of 107 patients (BI 46, AI 61) were enrolled. Compared with the BI group, the AI group had an earlier normal diet (19.83 h vs. 9.53 h, *p* < 0.001), ambulation (39.10 h vs. 12.70 h, *p* < 0.001), resumption of defecation (89.88 h vs. 27.90 h, *p* < 0.001), less need for additional analgesia (19.5% vs. 1.6%, *p* = 0.002) and shorter postoperative hospital stay (POS) (6.00 d vs. 1.91 d, *p* < 0.001) without increasing complications and readmission rates. Patients in the AI group had a median protocol score of 17 (IQR 16–18), and the compliance rate of the ERAS protocol was negatively correlated with the length of POS (*R*^2^ = 0.69, *p* < 0.001).

**Conclusions:**

The application of ERAS in pediatric LP is feasible and sustainable, with the potential for even greater impact as compliance improves. Common barriers were uncertain start time of surgery, lack of knowledge of ERAS among pathway participants, and support from anesthesiologists. Pre-determining the start time of surgery, strengthening preoperative education and positive communication among team members can help to promote the full implementation of ERAS program.

## Introduction

Enhanced recovery after surgery (ERAS) also known as Fast track surgery (FTS), was first proposed by Professor Henrik Keller in Denmark in 1977 (1), which is mainly based on the optimization of perioperative medical measures. It aims to reduce postoperative complications and stress response, shorten the length of hospital stay, reduce the risk of surgery, and promote postoperative recovery (2, 3).

Ureteropelvic junction obstruction (UPJO) is the most common cause of neonatal hydronephrosis. The total incidence rate was 1:500, and the male to female ratio was 2:1 (4). LP is the gold standard for the treatment of UPJO (5). Given the complexity of the procedure, postoperative recovery may be prolonged, with an average hospital stay of 4.20 days (6). Poor pain control and the presence of surgical drains may lead to prolonged recovery time (7). Slow recovery of bowel function may hinder oral fluid intake, reduce urinary washout of the urothelial epithelium, and increase the risk of bacterial attachment, which can trigger urinary tract infections and further prolong recovery. Overall, these factors cause great distress to patients and their families.

Our group previously published a pilot study of ERAS in pediatric laparoscopic pyeloplasty, which showed that implementation of ERAS was associated with shorter hospital stay (8). Although other studies on the application of ERAS in pediatric laparoscopic pyeloplasty have reached similar conclusions (9), they lack a clear control group and discuss the obstacles encountered in the implementation process. This article details the barriers we encountered in implementing ERAS in UPJO patients as well as the lessons learned.

## Methods

### Study population

The study was approved by the ethics committee at our institution under the relevant ethics approval number E2022140. We conducted a retrospective analysis of the database of pyeloplasty performed by the same surgeon from October 2008 to August 2021. The data included demographic characteristics and clinical data. Among them, from October 2018, our team leader, Chief physician Wang Dejuan, transferred to the current center and began to implement the ERAS program specifically for UPJO children, which was derived from the existing ERAS program and our practical experience.

### Similarities and differences between the ERAS and traditional protocols

Based on the existing ERAS protocols and our experience, an ERAS protocol specifically for UPJO children was developed and implemented, as shown in Table 1.

**Table 1:** Difference in perioperative care between the two groups.

ERAS item	Before implementation	After implementation
Preoperative consultation	Non-mandatory requirement	Mandatory requirements (Inform the ERAS management pathway)
Assessment of admission	Infection was excluded, and the diet of the children was not interfered	Infection and malnutrition were excluded, and nutritional support was given to those with malnutrition
Bowel preparation	Clean enema on the eve of surgery, and selective indwelling of gastrointestinal decompression tube and anal canal before surgery	On the night before surgery and on the morning of surgery, defecation was performed with kaiselu
Preoperative fasting	Patients were given a non-residue liquid diet for 1 day before surgery and fasted from 0:00 am on the day of surgery	Normal diet, breast milk, and electrolyte solution were allowed 6 h before anesthesia, 4 h before anesthesia, and 2 hours before anesthesia
Preemptive analgesia	No	NSAIDS suspension was administered orally at 8 PM the day before surgery
Prophylactic Antibiotics	30 min before the start of surgery	Yes (Same as the left)
Anesthesia	Conventional tracheal intubation, combined with intravenous general anesthesia	Conventional tracheal intubation, combined with intravenous general anesthesia (Medium-and short-acting anesthetics and muscle relaxants were used, and inhalation of anesthetics was stopped 30 min before surgery)
Intraoperative fluid management	Intravenous fluids were administered by monitoring factors such as blood pressure, heart rate, urine output, and central venous pressure	Yes (Same as the left)
Maintenance of normothermia	No	The ambient temperature of the operating room was increased, the nasopharyngeal temperature probe was used to monitor the body temperature, the warm blanket was used, and the intravenous infusion and irrigation fluid were heated
Wound infiltration anesthesia	No	Yes (ropivacaine injection: normal saline = 1:1)
Reginal analgesia	No	Yes (caudal anesthesia)
Gastric tube	A gastric tube was inserted after anesthesia and removed after awakening	No
Abdominal drainage tube	In general, the tube will be retained in situ for 3–5 days and subsequently removed if there is no obvious drainage fluid after 2 days	No
Urinary catheter	Leave for 6–7 days	Removed on the first day after surgery
Adjunctive analgesia	Infused tramadol intravenously	Administered NSAIDS suspension orally
Postoperative Diet	Fasting on the day of surgery, a small amount of plain boiled water was drunk on the first postoperative day, fluid diet was given for 2–3 days after surgery, a semiliquid diet was given for 3–5 days after surgery	After returning to the ward, the patients began to clear drink and gradually transitioned to a semi-liquid diet. On the first day after operation, if they could tolerate it, the normal diet was resumed
Postoperative activity nursing	No	Under the guidance of nursing, patients could sit up beside the bed or be carried by their parents 2 h after the operation, began to ambulate 6 h after the operation, and returned to normal activities on the first day after the operation

For children, parents are one of the key participants in ERAS programs. Surgeons and surgical ward nurses usually provide outpatient consultation and admission education to the patients’ families, introduce the ERAS management pathway and set up rehabilitation goals. Before surgery, the surgeon performs an infection and nutritional assessment to ensure that the patient is in good condition at the time of surgery. In addition, the following measures were taken before surgery: the use of Kaiselu laxative to avoid mechanical bowel preparation; The preoperative fasting time was minimized, and electrolyte beverages could be consumed up to 2 h before anesthesia.

In order to effectively control postoperative pain, multimodal methods have been used in analgesia: preemptive analgesia, caudal block analgesia, wound infiltration analgesia, and adjuvant oral analgesics. At the same time, intravenous-inhalation combined anesthesia should be given priority in the selection of anesthesia methods to reduce the dosage of drugs and shorten the time of anesthesia recovery. In addition, no gastrointestinal decompression was performed during the procedure, and normothermia was maintained by temperature monitoring and heated handling fluids.

**Figure 1:**
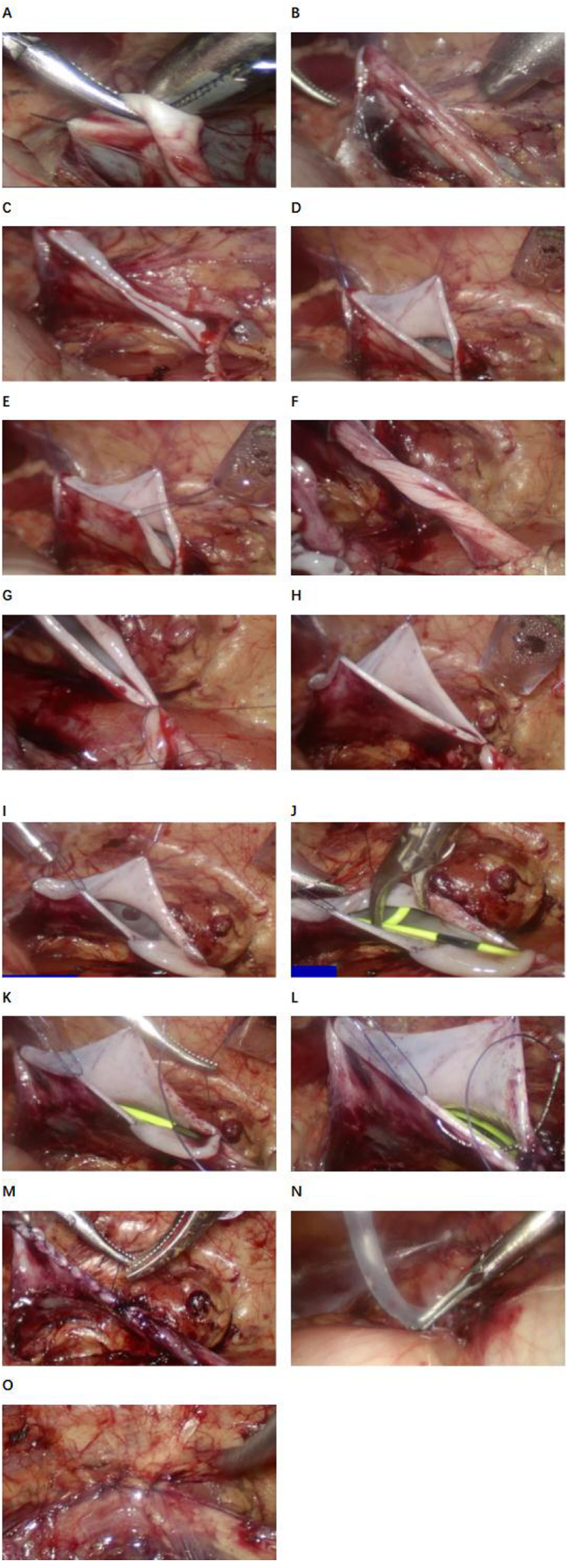
Procedure diagram of laparoscopic pyeloplasty. **(A)** First point suspension. **(B)** Effect after suspending the first point. **(C)** Cut the renal pelvis. **(D)** Second point suspension (anterior wall of renal pelvis). **(E)** Third point suspension (posterior wall of the renal pelvis). **(F)** Ureteral clipping. **(G)** Nadir suture. **(H)** Suspension at four o‘clock. **(I)** Suture of the posterior wall of the ureteropelvic anastomosis was completed. **(J)** Indwelling the DJ stent. **(K)** The posterior wall of the ureteropelvic anastomosis was sutured. **(L)** The rupture of the renal pelvis was sutured. **(M)** Complete suture (funnel type). **(N)** Abdominal drainage tube was placed in BI group. **(O)** Close the fascial break.

In order to achieve the goal of drainless management, the surgical technique was improved: the “no-touch” technique was used for suturing, and 5-0 micro-stick suture was used to fix the highest point of the cut renal pelvis, the left and right walls and the ureteropelvic rejunction. 18G trocar was used to pull out the end of the suture and fix it on the body surface of the renal pelvis (Figure 1). A DJ stent and urinary catheter were routinely placed, and an abdominal drainage tube was selectively placed. Sacral block was performed after the end of surgery.

After returning to the inpatient department, oral fluid intake and bedside activities could be gradually started. The oral analgesic dose was adjusted according to the visual analogue scale (VAS) (10). Children can be discharged when they are fully tolerant of oral intake and there is no sudden onset of severe pain or other conditions requiring hospitalization or observation.

The nurses followed up the children by telephone on the 1st, 3rd, and 7th day after discharge to check whether the children had critical symptoms. The patient was advised to return to the hospital if there were any signs of complications.

### Outcome

The primary outcome was the length of postoperative hospital stay (POS), and the secondary outcomes included the time to normal diet, time to active activity, time to bowel function recovery, VAS pain score (10), complication and readmission rate within 30 days (11). The implementation process was evaluated by the compliance of ERAS program.

### Statistical analysis

SPSS 26.0 statistical software (IBM SPSS Statistics for Windows; Armonk, NY, USA) performed statistical analysis. The measurement data were represented by mean ± standard deviation ($\bar{X}\pm SD$) if normal distribution and homogeneity of variance test were met. Otherwise, the median (lower quartile, upper quartile) [M (Q1, Q3)] was used. The Student's T test was used to compare the normal distribution measurement data between groups, and the Mann-Whitney U test was used to compare the non-normal distribution measurement data between groups. Chi-square test, continuity correction chi-square test (1 < T ≤ 5) and Fisher exact test (T ≤ 1) were used for statistical analysis of categorical data and categorical data. A two-tailed *p* value less than 0.05 was considered statistically significant. Bar charts and line graphs were used to depict the implementation of each ERAS program and the compliance of each patient, respectively. Pearson correlation test was used to analyze the relationship between ERAS compliance and POS.

## Results

A total of 116 children underwent elective laparoscopic pyeloplasty between 2008 and 2021, of whom 8 were excluded from this study due to a history of surgery on the affected kidney or upper ureter and 1 due to another urinary tract malformation (horseshoe kidney). A total of 107 patients were enrolled in this study, including 47 patients before (BI group) and 61 patients after (AI group) ERAS program. The baseline characteristics of the children are shown in Table 2. There were no significant differences in age, gender, weight, affected side and preoperative clinical manifestations between BI and AI groups.

**Table 2:** Patient demographics before and after ERAS implementation.

Characteristic	Before implementation	After implementation	*p*-value
Total patients, *n*	46	61	
Median age, years (IQR)	5.88 (1.75–9)	6 (2.17–9)	0.59
Sex: men/women, *n*	35/11	48/13	0.75
Side: right/left, *n*	34/12	41/20	0.45
Median Weight, kg (IQR)	18 (8.87–28.5)	22 (11.5–27.5)	0.56
Median Preoperative APD, mm (IQR)	37.5 (27.25–53.25)	29 (20–45)	0.06
Clinical presentation: Asymptomatic/symptomatic, *n*	34/12	44/17	0.84

IQR, interquartile range; APD, anteroposterior diameter of the renal pelvis.

The POS for the AI group was 1.91 (IQR 1–2.83) days, whereas for the BI group it was 6 (IQR 4.8–7.91) days, with the AI group significantly shorter than the BI group (*p* < 0.001). Compared with the BI group, the AI group had a shorter time to resume normal diet (19.83 h vs. 9.53 h, *p* < 0.001), ambulation time (39.10 h vs. 12.70 h, *p* < 0.001), intestinal function recovery time (89.88 h vs. 27.90 h, *p* < 0.001). When patients were assessed for pain on the first postoperative day, more patients in the BI group required additional analgesia than in the AI group (19.5% vs. 1.6%, *p* = 0.002). There was no significant difference between the two groups in overall 30-day surgery-related complications (17.3% vs. 6.5%, *p* = 0.079) or 30-day readmission rates (8.6% vs. 4.9%, *p* = 0.46). In terms of 30-day surgery-related complications, the most common complication was urinary tract infection (5 of 8 patients in the BI group and 1 of 4 patients in the AI group), followed by incomplete intestinal obstruction (2 of 8 patients in the BI group and 1 of 4 patients in the AI group), anastomotic stenosis (2 of 4 patients in the AI group), and other complications (1 of 4 patients in the AI group). Urinary extravasation (1 of 8 patients in the BI group) (Table 3).

**Table 3:** Outcomes of patients before and after ERAS implementation.

	Before implementation	After implementation	*p*-value
Median length of postoperative stay, days (IQR)	6 (4.8–7.91)	1.91 (1–2.83)	<0.001
Median start regular diet, hours (IQR)	19.83 (16–41.88)	9.53 (7.9–15.43)	<0.001
Median mobilization, hours (IQR)	39.1 (18.9–67.13)	12.7 (2.81–18.84)	<0.001
Median return of bowel function, hours (IQR)	89.88 (62.63–125.74)	27.9 (19.46–42.96)	<0.001
Postoperative pain score (VAS (10), >3, *n* (%)	9 (19.5%)	1 (1.6%)	0.002
Total complication within 30 days (11), *n* (%)	8 (17.3%)	4 (6.5%)	0.079
Readmissions within 30 days (11), *n* (%)	4 (8.6%)	3 (4.9%)	0.46

Start regular diet, time to start a semiliquid diet; mobilization, time to get out of bed; return of bowel function, time to start bowel movement; total complication within 30 days, complications associated with laparoscopic pyeloplasty; readmissions within 30 days, readmission due to surgery-related complications. IQR, interquartile range; APD, anteroposterior diameter of the renal pelvis; VAS, visual analogue scale.

**Figure 2:**
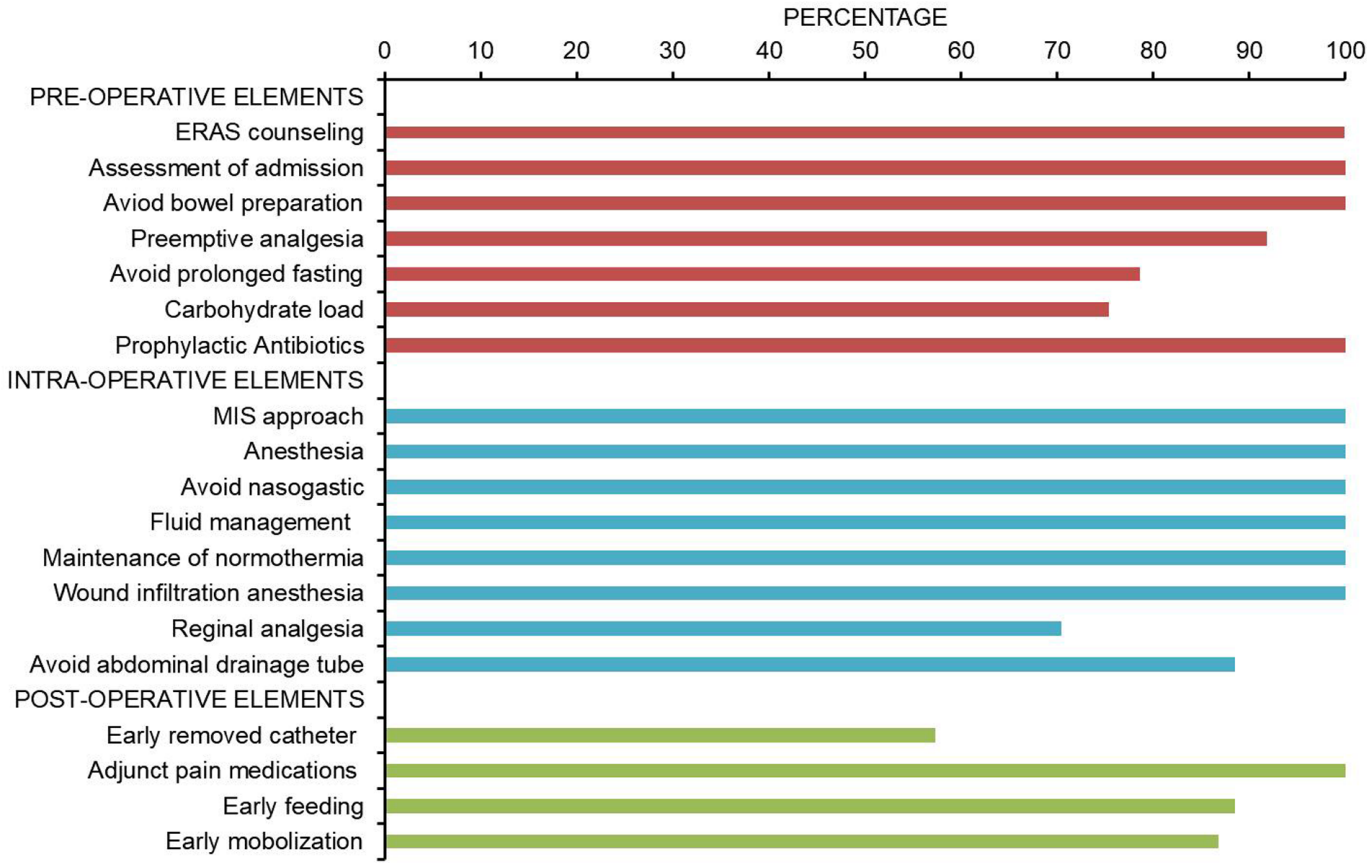
Pathway adherence. Compliance of ERAS program during the implementation of AI group (*n* = 61). The four items with the lowest compliance were early removal of urinary catheter (57.3%), sacral anesthesia (70.4%), shortening of preoperative fasting time (78.6%), and preoperative carbohydrate load (75.4%).

**Figure 3:**
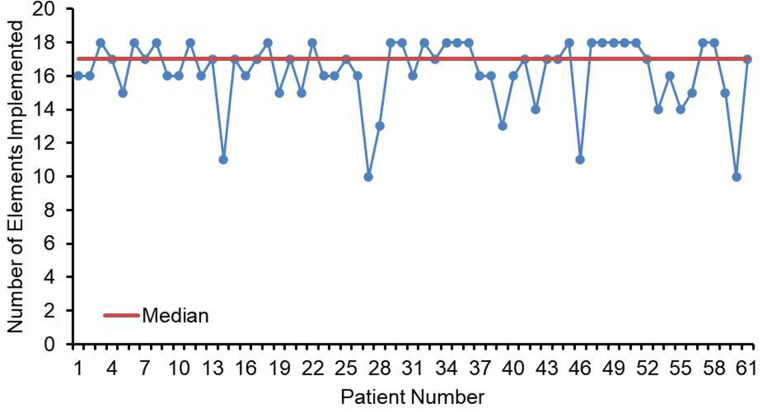
Line chart of elements implemented (*n* = 61). Numbers of ERAS items received for each child in the AI group. Median elements implemented is depicted by the orange line (IQR = 17).

**Figure 4:**
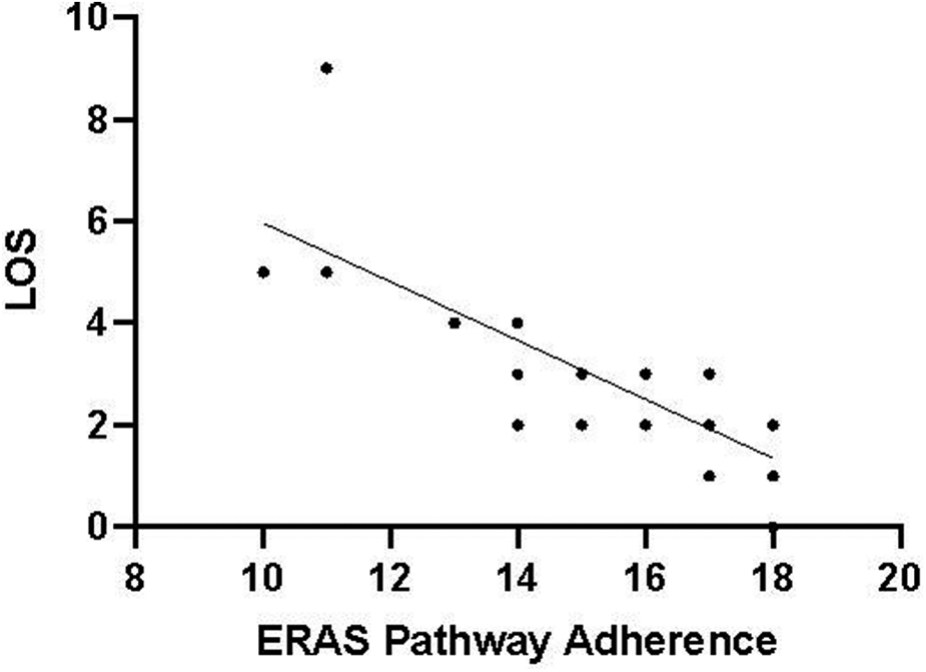
Protocol adherence for ERAS patients (*n* = 61) plotted against LOS. The LOS was negatively correlated with protocol adherence. The higher the compliance of ERAS, the shorter the postoperative hospital stay (*p* < 0.05).

The compliance of each ERAS item is shown in Figure 2, where the four items with lower than 80% compliance were early removal of urinary catheter, sacral anesthesia, shortened preoperative fasting time, and preoperative carbohydrate load. Patients in the AI group had a median protocol score of 17 (IQR 16–18) (Figure 3), and the protocol score was negatively correlated with the length of hospital stay (*R*^2^ = 0.69, *p* < 0.001) (Figure 4).

## Discussion

The potential benefits of ERAS in the perioperative care of pediatric urological patients have been confirmed, and similar to previous results (9, 12–14), the implementation of the ERAS protocol can significantly alleviate postoperative pain, promote early recovery of intestinal function and physical activity, and substantially shortened POS. Notably, there is a significant positive correlation between high compliance with the ERAS regimen and shortened POS, strongly suggesting that comprehensive and efficient implementation of the ERAS protocol can optimize the postoperative rehabilitation trajectory of patients, facilitating a smoother and faster physiological recovery process. In reviewing the obstacles and challenges in implementing our protocol, we found that measures such as minimizing preoperative fasting time, preoperative carbohydrate load, caudal block, and early urinary catheter removal were often easily hindered and ignored.

After a multidisciplinary effort, we developed an ERAS protocol for children undergoing LP that is continuously applied at our institution. After the protocol was developed, we became concerned about its sustainability in clinical practice. However, it seems impractical to fully perform or be accepted by patients for all ERAS programs during ERAS program implementation. In the preoperative phase, minimizing fasting time and carbohydrate load before surgery are often overlooked measures of ERAS, but neither of these measures has a compliance rate of 80% in our practice. The timing of the start of surgery affects the duration of preoperative fasting and the delivery of the carbohydrate load. For the first operation, the duration of anesthesia can be predetermined, so that the corresponding plan can be executed accurately. For subsequent surgery, however, the timing of the onset of anesthesia can only be estimated, and patients may be asked to fast for a longer period of time before surgery to avoid disrupting the sequence of surgery or cancelation of surgery. Determining the start time of surgery or setting the LP procedure as the first procedure helps to achieve accurate preoperative fasting and carbohydrate loading.

In China, patients requiring surgery typically undergo preoperative examinations after admission, which may take several days. Therefore, the total hospital stay in our study may not be suitable for assessing patient recovery status. In a large cohort study conducted in Beijing from July 2016 to July 2018 involving 279 pediatric patients undergoing LP, the average POS was 6 days (range 3–16 days), which is significantly longer than in our study (11). In contrast, studies from the United States and Canada reported total hospital stays of 1.4 ± 0.5 days and 1.1 (IQR 1.1) days, respectively (6, 9). In our study, the POS was 1.91 (IQR 1–2.83) days. Clearly, there are significant differences between China and North America, likely due to different discharge criteria and cultural differences. In China, we have more conservative discharge criteria, and parents generally do not accept discharge on the day of surgery. Nevertheless, our ERAS protocol has indeed enhanced postoperative recovery and significantly shortened the hospital stay.

Pain is the most common reason for prolonged hospital stay and readmission (7, 15). Similar to the analgesic regimens of other institutions (16), multimodal analgesia was adopted in this study, including preemptive analgesia, wound infiltration anesthesia, regional nerve block and oral NSAIS as adjunctive analgesia, which effectively reduced postoperative pain in children. Caudal block is the key to postoperative analgesia (8), but the compliance rate is less than 80%, which may be affected by factors such as the anesthesiologist's personal preference, skill level and equipment. Lack of support from anesthesiologists is a common obstacle in the implementation of ERAS (17). This practice was not widely performed before the transfer of our team leader to the current center. The implementation of ERAS does require the multidisciplinary team to make changes in perioperative management. Therefore, it is necessary to actively organize inter-professional meetings to promote communication between pathway participants, enhance the awareness of implementing ERAS and improve the technical level.

For LP, there is currently controversy about the optimal use and indwelling time of the tube, which is closely related to LOS (9, 18). Studies conducted by Vinodh Murali and Donati-Bourne et al. showed that day discharge could be successfully achieved without postoperative drainage and early removal of urinary catheters in adult patients undergoing LP surgery, indicating that early urinary catheterless management is a feasible approach (7, 15). This was also found to be associated with shorter LOS in our previous report (8). Early urinary catheter removal is one of the measures that we are most likely to encounter obstacles in the implementation of ERAS. We routinely place a DJ stent during surgery and perform a sacral block after surgery. Urinary catheter is used for bladder decompression to prevent urinary tract infections (19, 20). In the case of older children, we need to ensure that they can remove the urinary catheter after voluntary active urination, which is somewhat challenging for us. Because parents often believe that long term bed rest is necessary after complex surgery, we need to strengthen preoperative education to make them understand the benefits of ERAS implementation, reduce anxiety and help overcome the fear of children, so as to obtain their cooperation.

Other participants in the ERAS pathway, such as rotation doctors or nurses, lack of understanding of the pathway is also one of the obstacles in the implementation of ERAS (21). Therefore, the use of standardized medical orders and the participation of specialist urological residents and nursing instructors can reduce the changes in the implementation of postoperative ERAS measures during the rotation of new residents and nurses.

Our study has some limitations; the implementation and effectiveness results are only applicable to our institution and cannot be fully generalized to other centers. No direct inferences can be drawn about the effects of the interventions studied on the established outcomes because randomization was not performed but rather patients were directly compared before and after ERAS implementation. ERAS protocol contains many complex items, and it is difficult to determine which measures are most critical for reducing LOS and postoperative prognosis. This article mainly focuses on ERP implementation and is not a comprehensive assessment of patient outcomes. We successfully implemented an ERAS protocol in pediatric LP and improved the recovery process.

In conclusion, the application of ERAS in pediatric LP is feasible and sustainable, and the effect will be more significant with the improvement of compliance. Common barriers were uncertain start time of surgery, lack of knowledge of ERAS among pathway participants, and support from anesthesiologists. Pre-determining the start time of surgery, strengthening perioperative education and active communication for family members to obtain the support of anesthesiologists are helpful to promote the full implementation of ERAS program. We summarize institutional experiences in the hope of providing insights to others interested in ERAS implementation.

## Data Availability

The original contributions presented in the study are included in the article/Supplementary Material, further inquiries can be directed to the corresponding authors.
